# (*E*)-9-(4-Fluoro­styr­yl)-3,3,6,6-tetra­methyl-3,4,5,6,7,9-hexa­hydro-2*H*-xanthene-1,8-dione

**DOI:** 10.1107/S1600536813014049

**Published:** 2013-05-31

**Authors:** Jae Kyun Lee, Sun-Joon Min, Yong Seo Cho, Joo Hwan Cha, Sung Ok Won

**Affiliations:** a Center for Neuro-Medicine, Korea Institute of Science & Technology, Hwarangro 14-gil, Seongbuk-gu, Seoul 136-791, Republic of Korea; b Advanced Analysis Center, Korea Institute of Science & Technology, Hwarangro 14-gil, Seongbuk-gu, Seoul 136-791, Republic of Korea

## Abstract

In the title compound, C_25_H_27_FO_3_, each of the cyclo­hexenone rings adopts a half-chair conformation, whereas the six-membered pyran ring adopts a flattened boat conformation, with the O and methine C atoms deviating by 0.0769 (15) and 0.196 (2) Å, respectively, from the plane of the other four atoms (r.m.s. deviation = 0.004 Å). The C=C double bond adopts an *E* conformation. The dihedral angle between the benzene and pyran (all atoms) rings is 89.94 (10)°. In the crystal, weak C—H⋯O hydrogen bonds link the mol­ecules into chains running parallel to the *b* axis.

## Related literature

For the crystal structures of xanthenes derivatives studied recently by our group, see: Cha *et al.* (2012 ▶, 2013 ▶); Lee *et al.* (2012 ▶).
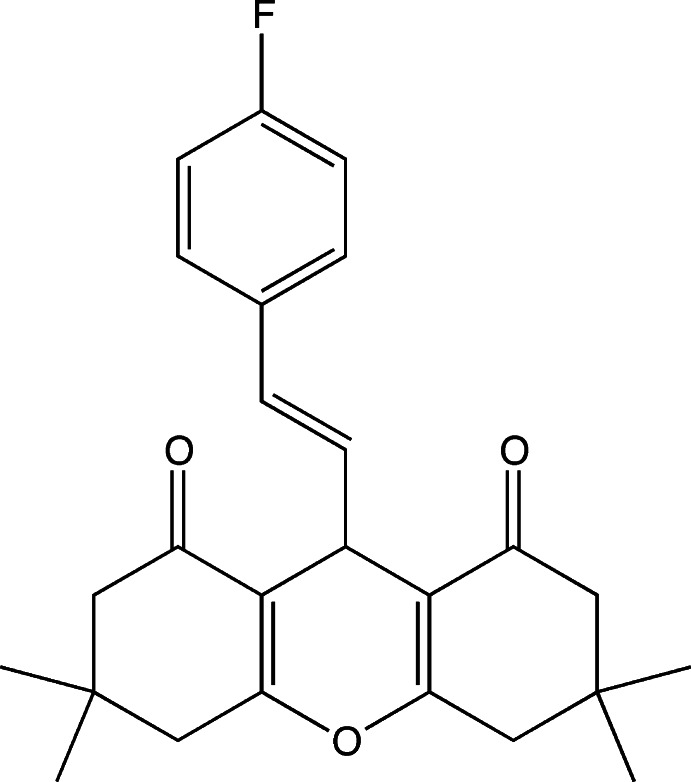


## Experimental

### 

#### Crystal data

C_25_H_27_FO_3_
*M* *_r_* = 394.48
Monoclinic, 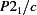

*a* = 5.9367 (7) Å
*b* = 18.8521 (16) Å
*c* = 19.3709 (16) Å
β = 99.681 (3)°
*V* = 2137.1 (4) Å^3^
*Z* = 4
Mo *K*α radiation
μ = 0.09 mm^−1^
*T* = 296 K
0.30 × 0.20 × 0.20 mm

#### Data collection

Rigaku R-AXIS RAPID diffractometer
Absorption correction: multi-scan (*ABSCOR*; Rigaku, 1995 ▶) *T*_min_ = 0.773, *T*_max_ = 0.983
20480 measured reflections
4864 independent reflections
2857 reflections with *F* ^2^ > 2σ(*F* ^2^)
*R*_int_ = 0.037

#### Refinement

*R*[*F* ^2^ > 2σ(*F* ^2^)] = 0.053
*wR*(*F* ^2^) = 0.169
*S* = 1.09
4864 reflections
274 parameters
H atoms treated by a mixture of independent and constrained refinement
Δρ_max_ = 0.42 e Å^−3^
Δρ_min_ = −0.24 e Å^−3^

### 

Data collection: *RAPID-AUTO* (Rigaku, 2006 ▶); cell refinement: *RAPID-AUTO*; data reduction: *RAPID-AUTO*; program(s) used to solve structure: *Il Milione* (Burla *et al.*, 2007 ▶); program(s) used to refine structure: *SHELXL97* (Sheldrick, 2008 ▶); molecular graphics: *CrystalStructure* (Rigaku, 2010 ▶); software used to prepare material for publication: *CrystalStructure*.

## Supplementary Material

Crystal structure: contains datablock(s) global, I. DOI: 10.1107/S1600536813014049/ff2106sup1.cif

Structure factors: contains datablock(s) I. DOI: 10.1107/S1600536813014049/ff2106Isup2.hkl

Supplementary material file. DOI: 10.1107/S1600536813014049/ff2106Isup3.cml

Additional supplementary materials:  crystallographic information; 3D view; checkCIF report

## Figures and Tables

**Table 1:** Hydrogen-bond geometry (Å, °)

*D*—H⋯*A*	*D*—H	H⋯*A*	*D*⋯*A*	*D*—H⋯*A*
C22—H22*B*⋯O2^i^	0.96	2.60	3.533 (4)	163

Symmetry code: (i) 
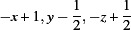
.

**Fig. 1.**
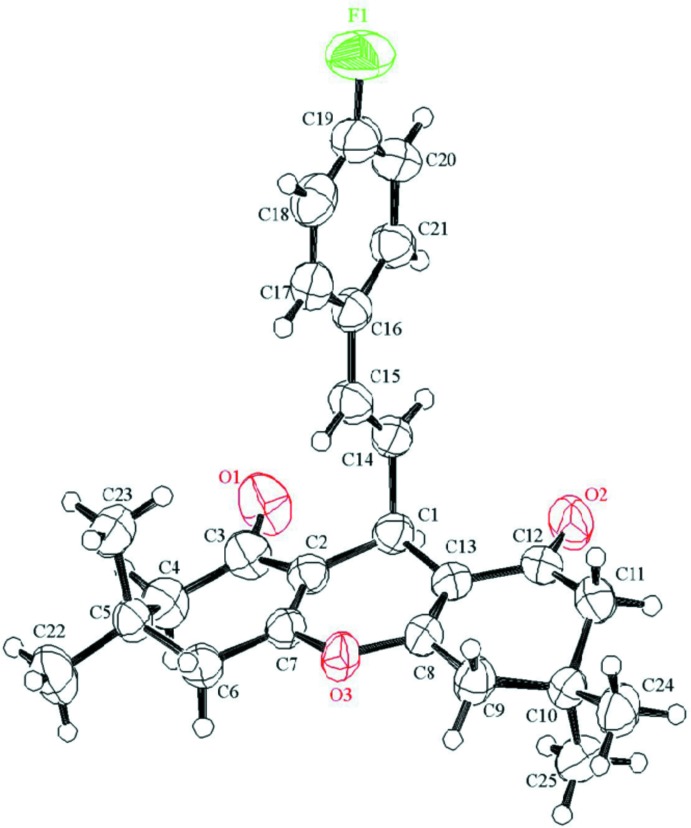
The molecular structure of (I) showing the atomic numbering and 50% probability displacement ellipsoid.

## supplementary crystallographic information

### Comment

As part of our ongoing study of the substituent effect on the solid state structures of xanthene derivatives (Cha *et al.*, 2012; Lee *et al.*, 2012), we present here the crystal structure of the title compound (I) (Fig. 1).

The starting material, (*E*)-2.2-(3-(4-Fluorophenyl)prop-2-ene-1,1-diyl) bis(3-hydroxy-5,5-δimethylcyclohex-2-enone) was prepared according to the reported method (Cha *et al.*, 2013). In (I) (Fig. 1), the bond lengths and angles are normal and correspond to those observed in related structures (Cha *et al.*, 2013). The dihedral angle between the benzene ring (C16 - C21) system and the pyran ring (C1—C2—C7—O3—C8—C13) is 89.94 (10)°. The C14=C15 double bond has an *E* conformation. All two cyclohexenone rings in (Fig. 1) display half-chair conformation, whereas the pyran ring adopts a boat conformation.

In the crystal, weak intermolecular C—H···O hydrogen bonds link molecules into chains running parallel to the *b* axis.

### Experimental

To solution of (*E*)-2.2-(3-(4-Fluorophenyl)prop-2-ene-1,1-diyl)bis (3-hydroxy-5,5-dimethylcyclohex-2-enone) (1.25 mmol) was added methanol and catalytic amounts of sulfuric acid in under nitrogen atmosphere. After stirring for 4 h, the progress of reaction was monitored by TLC. The solvent was evaporated and the remaining residue dissolved in ethyl acetate. The mixture was neutralized with saturated sodium bicarbonate and the solution was extracted with ethyl acetate. The resulting residue solid was purified by recrystallization from ethanol and methylene chloride to afford yield 91%) colourless block type crystals suitable for X-ray analysis.

### Refinement

All hydrogen atoms were positioned geometrically and refined using a riding model with C—H = 0.93–0.98 Å and *U*iso(H) = 1.2 or 1.5 *U*eq(C).

### Crystal data

**Table:**

C_25_H_27_FO_3_	*F*(000) = 840.00
*M**_r_* = 394.48	*D*_x_ = 1.226 Mg m^−^^3^
Monoclinic, *P*2_1_/*c*	Mo *K*α radiation, λ = 0.71075 Å
Hall symbol: -P 2ybc	Cell parameters from 12751 reflections
*a* = 5.9367 (7) Å	θ = 3.0–27.5°
*b* = 18.8521 (16) Å	µ = 0.09 mm^−^^1^
*c* = 19.3709 (16) Å	*T* = 296 K
β = 99.681 (3)°	Chunk, colourless
*V* = 2137.1 (4) Å^3^	0.30 × 0.20 × 0.20 mm
*Z* = 4	

### Data collection

**Table:**

Rigaku R-AXIS RAPID diffractometer	2857 reflections with *F*^2^ > 2σ(*F*^2^)
Detector resolution: 10.000 pixels mm^-1^	*R*_int_ = 0.037
ω scans	θ_max_ = 27.5°
Absorption correction: multi-scan (*ABSCOR*; Rigaku, 1995)	*h* = −7→7
*T*_min_ = 0.773, *T*_max_ = 0.983	*k* = −24→24
20480 measured reflections	*l* = −22→25
4864 independent reflections	

### Refinement

**Table:**

Refinement on *F*^2^	Secondary atom site location: difference Fourier map
*R*[*F*^2^ > 2σ(*F*^2^)] = 0.053	Hydrogen site location: inferred from neighbouring sites
*wR*(*F*^2^) = 0.169	H atoms treated by a mixture of independent and constrained refinement
*S* = 1.09	*w* = 1/[σ^2^(*F*_o_^2^) + (0.0774*P*)^2^ + 0.3562*P*] where *P* = (*F*_o_^2^ + 2*F*_c_^2^)/3
4864 reflections	(Δ/σ)_max_ < 0.001
274 parameters	Δρ_max_ = 0.42 e Å^−^^3^
0 restraints	Δρ_min_ = −0.24 e Å^−^^3^
Primary atom site location: structure-invariant direct methods	

### Special details

**Table:**

Geometry. All e.s.d.'s (except the e.s.d. in the dihedral angle between two l.s. planes) are estimated using the full covariance matrix. The cell e.s.d.'s are taken into account individually in the estimation of e.s.d.'s in distances, angles and torsion angles; correlations between e.s.d.'s in cell parameters are only used when they are defined by crystal symmetry. An approximate (isotropic) treatment of cell e.s.d.'s is used for estimating e.s.d.'s involving l.s. planes.
Refinement. Refinement was performed using all reflections. The weighted *R*-factor (*wR*) and goodness of fit (*S*) are based on *F*^2^. *R*-factor (gt) are based on *F*. The threshold expression of *F*^2^ > 2.0 σ(*F*^2^) is used only for calculating *R*-factor (gt).

### Fractional atomic coordinates and isotropic or equivalent isotropic displacement parameters (Å^2^)

**Table:**

	*x*	*y*	*z*	*U*_iso_*/*U*_eq_	
F1	1.3085 (4)	0.41875 (10)	0.55088 (8)	0.1071 (7)	
O1	0.3415 (3)	0.16938 (9)	0.26856 (10)	0.0755 (6)	
O2	0.5001 (4)	0.40605 (8)	0.14354 (9)	0.0743 (6)	
O3	0.9010 (3)	0.19315 (7)	0.12327 (7)	0.0508 (4)	
C1	0.5863 (4)	0.26673 (10)	0.19791 (9)	0.0457 (5)	
C2	0.6201 (4)	0.18746 (10)	0.19814 (9)	0.0427 (5)	
C3	0.4790 (4)	0.14307 (12)	0.23554 (11)	0.0541 (6)	
C4	0.5029 (4)	0.06354 (12)	0.22949 (12)	0.0591 (6)	
C5	0.7461 (4)	0.03924 (10)	0.22594 (10)	0.0451 (5)	
C6	0.8316 (4)	0.07994 (10)	0.16669 (10)	0.0484 (5)	
C7	0.7745 (4)	0.15633 (10)	0.16515 (9)	0.0424 (5)	
C8	0.8474 (4)	0.26351 (10)	0.10936 (9)	0.0449 (5)	
C9	0.9782 (4)	0.29226 (11)	0.05617 (10)	0.0517 (5)	
C10	0.8696 (4)	0.35891 (10)	0.01931 (9)	0.0469 (5)	
C11	0.7974 (5)	0.40786 (11)	0.07479 (11)	0.0592 (6)	
C12	0.6512 (4)	0.37327 (10)	0.12129 (10)	0.0512 (6)	
C13	0.6981 (4)	0.29901 (10)	0.14075 (9)	0.0445 (5)	
C14	0.6839 (4)	0.30007 (11)	0.26838 (10)	0.0487 (5)	
C15	0.8830 (4)	0.28526 (11)	0.30532 (11)	0.0501 (5)	
C16	0.9898 (4)	0.31916 (10)	0.37108 (10)	0.0454 (5)	
C17	1.2137 (4)	0.30241 (11)	0.40059 (11)	0.0542 (6)	
C18	1.3216 (5)	0.33576 (13)	0.46122 (12)	0.0635 (6)	
C19	1.2036 (5)	0.38498 (13)	0.49183 (12)	0.0671 (7)	
C20	0.9810 (5)	0.40299 (12)	0.46566 (12)	0.0638 (7)	
C21	0.8770 (4)	0.36963 (11)	0.40537 (11)	0.0539 (6)	
C22	0.7571 (6)	−0.03946 (12)	0.21348 (15)	0.0782 (8)	
C23	0.8977 (5)	0.05692 (13)	0.29641 (11)	0.0641 (7)	
C24	1.0446 (5)	0.39614 (12)	−0.01757 (12)	0.0632 (7)	
C25	0.6614 (5)	0.33915 (13)	−0.03464 (11)	0.0621 (6)	
H1	0.4221	0.2767	0.1876	0.0549*	
H4A	0.4023	0.0474	0.1877	0.0710*	
H4B	0.4533	0.0413	0.2695	0.0710*	
H6A	0.9961	0.0747	0.1720	0.0581*	
H6B	0.7652	0.0589	0.1222	0.0581*	
H9A	0.9885	0.2560	0.0213	0.0620*	
H9B	1.1323	0.3034	0.0790	0.0620*	
H11A	0.9339	0.4264	0.1038	0.0711*	
H11B	0.7144	0.4478	0.0513	0.0711*	
H17	1.2925	0.2683	0.3794	0.0651*	
H18	1.4716	0.3246	0.4804	0.0762*	
H20	0.9033	0.4365	0.4879	0.0766*	
H21	0.7266	0.3812	0.3869	0.0646*	
H22A	0.6699	−0.0508	0.1685	0.0938*	
H22B	0.6951	−0.0643	0.2493	0.0938*	
H22C	0.9133	−0.0534	0.2148	0.0938*	
H23A	0.8403	0.0328	0.3335	0.0769*	
H23B	0.8958	0.1072	0.3043	0.0769*	
H23C	1.0515	0.0418	0.2954	0.0769*	
H24A	0.9776	0.4381	−0.0405	0.0758*	
H24B	1.0908	0.3647	−0.0516	0.0758*	
H24C	1.1756	0.4090	0.0163	0.0758*	
H25A	0.5517	0.3152	−0.0118	0.0745*	
H25B	0.7074	0.3084	−0.0693	0.0745*	
H25C	0.5940	0.3814	−0.0568	0.0745*	
H14	0.606 (5)	0.3358 (14)	0.2853 (13)	0.080 (8)*	
H15	0.976 (5)	0.2514 (13)	0.2866 (13)	0.070 (8)*	

### Atomic displacement parameters (Å^2^)

**Table:**

	*U*^11^	*U*^22^	*U*^33^	*U*^12^	*U*^13^	*U*^23^
F1	0.1157 (15)	0.1169 (14)	0.0759 (10)	−0.0103 (11)	−0.0212 (10)	−0.0327 (10)
O1	0.0723 (12)	0.0670 (11)	0.1011 (13)	0.0029 (9)	0.0546 (11)	−0.0011 (9)
O2	0.1033 (15)	0.0543 (10)	0.0726 (11)	0.0236 (9)	0.0359 (10)	0.0042 (8)
O3	0.0574 (9)	0.0486 (8)	0.0522 (8)	0.0101 (7)	0.0257 (7)	0.0112 (7)
C1	0.0488 (13)	0.0489 (11)	0.0410 (10)	0.0065 (9)	0.0121 (9)	−0.0005 (9)
C2	0.0447 (12)	0.0447 (11)	0.0398 (10)	0.0003 (9)	0.0109 (8)	−0.0013 (8)
C3	0.0488 (13)	0.0572 (13)	0.0605 (13)	−0.0001 (10)	0.0210 (11)	−0.0010 (10)
C4	0.0537 (14)	0.0595 (14)	0.0660 (14)	−0.0074 (11)	0.0149 (11)	0.0005 (11)
C5	0.0478 (12)	0.0414 (10)	0.0488 (11)	−0.0023 (9)	0.0157 (9)	0.0013 (9)
C6	0.0528 (13)	0.0471 (11)	0.0486 (11)	0.0026 (9)	0.0183 (10)	0.0003 (9)
C7	0.0451 (12)	0.0461 (11)	0.0380 (9)	0.0001 (9)	0.0128 (8)	0.0019 (8)
C8	0.0515 (13)	0.0440 (11)	0.0394 (10)	0.0016 (9)	0.0084 (9)	0.0041 (8)
C9	0.0548 (13)	0.0534 (12)	0.0495 (11)	0.0022 (10)	0.0162 (10)	0.0080 (10)
C10	0.0560 (13)	0.0445 (11)	0.0399 (10)	−0.0030 (9)	0.0073 (9)	0.0033 (9)
C11	0.0814 (18)	0.0426 (11)	0.0550 (12)	−0.0065 (11)	0.0153 (12)	−0.0009 (10)
C12	0.0691 (15)	0.0439 (11)	0.0413 (10)	0.0030 (10)	0.0112 (10)	−0.0063 (9)
C13	0.0527 (13)	0.0450 (10)	0.0364 (9)	0.0026 (9)	0.0100 (9)	−0.0000 (8)
C14	0.0591 (14)	0.0477 (11)	0.0412 (10)	0.0116 (10)	0.0143 (10)	−0.0020 (9)
C15	0.0520 (14)	0.0481 (12)	0.0519 (12)	0.0063 (10)	0.0133 (10)	−0.0070 (10)
C16	0.0503 (13)	0.0432 (10)	0.0444 (10)	0.0008 (9)	0.0124 (9)	0.0033 (9)
C17	0.0538 (14)	0.0541 (12)	0.0558 (12)	0.0031 (10)	0.0121 (10)	0.0050 (10)
C18	0.0553 (15)	0.0706 (15)	0.0611 (14)	−0.0025 (12)	0.0000 (11)	0.0090 (12)
C19	0.0787 (19)	0.0662 (15)	0.0524 (13)	−0.0144 (14)	−0.0006 (12)	−0.0048 (12)
C20	0.0752 (18)	0.0623 (14)	0.0541 (12)	0.0026 (12)	0.0113 (12)	−0.0123 (11)
C21	0.0537 (14)	0.0561 (13)	0.0521 (12)	0.0046 (10)	0.0098 (10)	−0.0038 (10)
C22	0.100 (3)	0.0492 (13)	0.0961 (19)	−0.0046 (13)	0.0465 (17)	−0.0001 (13)
C23	0.0675 (17)	0.0670 (15)	0.0568 (13)	−0.0014 (12)	0.0076 (12)	0.0103 (12)
C24	0.0704 (17)	0.0602 (14)	0.0603 (13)	−0.0066 (12)	0.0152 (12)	0.0136 (11)
C25	0.0646 (16)	0.0717 (15)	0.0486 (12)	−0.0028 (12)	0.0057 (11)	0.0026 (11)

### Geometric parameters (Å, º)

**Table:**

F1—C19	1.365 (3)	C19—C20	1.376 (4)
O1—C3	1.224 (3)	C20—C21	1.378 (3)
O2—C12	1.226 (3)	C1—H1	0.980
O3—C7	1.382 (3)	C4—H4A	0.970
O3—C8	1.380 (3)	C4—H4B	0.970
C1—C2	1.508 (3)	C6—H6A	0.970
C1—C13	1.511 (3)	C6—H6B	0.970
C1—C14	1.526 (3)	C9—H9A	0.970
C2—C3	1.460 (3)	C9—H9B	0.970
C2—C7	1.338 (3)	C11—H11A	0.970
C3—C4	1.512 (4)	C11—H11B	0.970
C4—C5	1.527 (4)	C14—H14	0.91 (3)
C5—C6	1.536 (3)	C15—H15	0.95 (3)
C5—C22	1.506 (3)	C17—H17	0.930
C5—C23	1.540 (3)	C18—H18	0.930
C6—C7	1.479 (3)	C20—H20	0.930
C8—C9	1.492 (3)	C21—H21	0.930
C8—C13	1.336 (3)	C22—H22A	0.960
C9—C10	1.533 (3)	C22—H22B	0.960
C10—C11	1.531 (3)	C22—H22C	0.960
C10—C24	1.527 (4)	C23—H23A	0.960
C10—C25	1.524 (3)	C23—H23B	0.960
C11—C12	1.501 (4)	C23—H23C	0.960
C12—C13	1.464 (3)	C24—H24A	0.960
C14—C15	1.305 (3)	C24—H24B	0.960
C15—C16	1.470 (3)	C24—H24C	0.960
C16—C17	1.392 (3)	C25—H25A	0.960
C16—C21	1.394 (3)	C25—H25B	0.960
C17—C18	1.390 (3)	C25—H25C	0.960
C18—C19	1.358 (4)		
			
F1···C21	3.597 (3)	C1···H9B^ii^	3.3111
O1···C1	2.832 (3)	C3···H6A^ii^	3.1946
O1···C7	3.523 (3)	C3···H23C^ii^	3.5247
O1···C14	3.194 (3)	C4···H6A^ii^	3.0348
O2···C1	2.844 (3)	C4···H23C^ii^	3.1830
O2···C8	3.517 (3)	C6···H4A^iv^	3.4001
O2···C14	3.185 (3)	C6···H20^xi^	3.5732
O3···C1	2.900 (3)	C8···H1^iv^	3.5015
C2···C5	2.919 (3)	C8···H18^v^	3.4780
C2···C8	2.759 (3)	C9···H1^iv^	3.3551
C2···C15	3.009 (3)	C11···H23A^vi^	3.4709
C2···C23	3.367 (3)	C11···H23C^vi^	3.5698
C3···C6	2.918 (4)	C11···H24A^xii^	3.3105
C3···C14	3.222 (3)	C12···H9B^ii^	3.3236
C3···C23	3.036 (4)	C12···H24C^ii^	3.2611
C4···C7	2.809 (4)	C13···H9B^ii^	3.3720
C7···C13	2.755 (3)	C14···H17^ii^	3.4730
C7···C14	3.463 (3)	C14···H22B^iii^	3.3846
C7···C23	3.144 (3)	C15···H22C^vi^	3.3203
C8···C11	2.806 (3)	C15···H25B^ix^	3.3106
C8···C14	3.451 (3)	C16···H9A^ix^	3.2382
C8···C25	3.162 (3)	C16···H22A^vi^	3.3468
C9···C12	2.918 (4)	C16···H22C^vi^	3.0316
C10···C13	2.941 (3)	C16···H25B^ix^	3.2531
C12···C14	3.142 (3)	C17···H9A^ix^	3.0860
C12···C25	3.099 (3)	C17···H21^iv^	3.4376
C13···C15	3.203 (3)	C17···H22A^vi^	3.1996
C13···C25	3.452 (3)	C17···H22C^vi^	3.5190
C14···C21	3.009 (3)	C17···H24B^ix^	3.3980
C16···C19	2.761 (3)	C17···H25A^i^	3.2675
C17···C20	2.771 (4)	C17···H25B^i^	3.5667
C18···C21	2.754 (4)	C17···H14^iv^	3.54 (3)
F1···C4^i^	3.474 (3)	C18···H9A^ix^	3.0056
F1···C6^i^	3.512 (3)	C18···H21^iv^	3.1224
F1···C7^i^	3.535 (3)	C18···H22A^vi^	3.3064
O1···O3^ii^	3.534 (3)	C18···H25A^i^	3.1618
O1···C23^ii^	3.494 (4)	C19···H9A^ix^	3.0437
O2···C22^iii^	3.533 (4)	C19···H20^x^	3.4586
O3···O1^iv^	3.534 (3)	C19···H22A^vi^	3.5298
C4···F1^v^	3.474 (3)	C20···H6B^ix^	3.5580
C6···F1^v^	3.512 (3)	C20···H9A^ix^	3.1827
C7···F1^v^	3.535 (3)	C20···H18^ii^	3.4206
C16···C22^vi^	3.588 (4)	C20···H20^x^	3.1976
C22···O2^vii^	3.533 (4)	C21···H9A^ix^	3.2544
C22···C16^viii^	3.588 (4)	C21···H18^ii^	3.1313
C23···O1^iv^	3.494 (4)	C21···H22A^vi^	3.5814
F1···H18	2.5290	C21···H22C^vi^	3.1694
F1···H20	2.5297	C21···H25B^ix^	3.5623
O1···H1	2.6521	C22···H21^vii^	3.5200
O1···H4A	2.8387	C22···H14^vii^	3.19 (3)
O1···H4B	2.5043	C23···H4B^iv^	3.4377
O1···H23B	3.4520	C23···H11A^viii^	3.1848
O1···H14	3.50 (3)	C23···H24A^ix^	3.1156
O2···H1	2.6498	C23···H24B^ix^	3.3204
O2···H11A	2.8347	C24···H11B^xii^	3.3831
O2···H11B	2.4886	C24···H23A^xi^	3.2261
O2···H25A	3.5198	C24···H23B^xi^	3.4164
O2···H14	3.02 (3)	C24···H24A^xii^	3.3320
O3···H6A	2.4523	C24···H25A^iv^	3.3600
O3···H6B	2.6557	C24···H25C^iv^	3.4827
O3···H9A	2.4322	C25···H17^v^	3.2327
O3···H9B	2.7096	C25···H18^v^	3.3155
O3···H15	3.31 (3)	C25···H24B^ii^	3.3827
C1···H15	2.65 (3)	C25···H24C^ii^	3.4616
C2···H4A	2.9317	H1···O3^ii^	3.5083
C2···H4B	3.3091	H1···C8^ii^	3.5015
C2···H6A	3.1832	H1···C9^ii^	3.3551
C2···H6B	3.0345	H1···H9B^ii^	2.5347
C2···H23B	2.8394	H1···H22B^iii^	3.3533
C2···H14	3.27 (3)	H1···H15^ii^	3.5543
C2···H15	2.76 (3)	H4A···F1^v^	2.6922
C3···H1	2.6867	H4A···C6^ii^	3.4001
C3···H6B	3.3883	H4A···H6A^ii^	2.4346
C3···H23A	3.3393	H4A···H21^vii^	3.4827
C3···H23B	2.6914	H4A···H22C^ii^	3.5817
C3···H15	3.59 (3)	H4A···H23C^ii^	3.1875
C4···H6A	3.3095	H4B···O2^vii^	3.0419
C4···H6B	2.7999	H4B···C23^ii^	3.4377
C4···H22A	2.7237	H4B···H6A^ii^	3.0997
C4···H22B	2.6671	H4B···H23C^ii^	2.5189
C4···H22C	3.3330	H6A···F1^xi^	3.2287
C4···H23A	2.6551	H6A···O1^iv^	3.0975
C4···H23B	2.6606	H6A···C3^iv^	3.1946
C4···H23C	3.3172	H6A···C4^iv^	3.0348
C6···H4A	2.7173	H6A···H4A^iv^	2.4346
C6···H4B	3.3234	H6A···H4B^iv^	3.0997
C6···H22A	2.6471	H6A···H20^xi^	3.5224
C6···H22B	3.3240	H6B···F1^v^	2.8599
C6···H22C	2.6962	H6B···C20^xi^	3.5580
C6···H23A	3.3434	H6B···H20^xi^	2.8574
C6···H23B	2.6773	H9A···C16^xi^	3.2382
C6···H23C	2.7096	H9A···C17^xi^	3.0860
C7···H1	3.1660	H9A···C18^xi^	3.0056
C7···H4A	3.1005	H9A···C19^xi^	3.0437
C7···H23B	2.8257	H9A···C20^xi^	3.1827
C7···H23C	3.5123	H9A···C21^xi^	3.2544
C7···H15	3.04 (3)	H9A···H17^xi^	3.5637
C8···H1	3.1658	H9A···H18^v^	3.3955
C8···H11A	3.1173	H9A···H18^xi^	3.4535
C8···H25A	2.8550	H9B···O2^iv^	3.0258
C8···H25B	3.5231	H9B···C1^iv^	3.3111
C8···H15	3.40 (3)	H9B···C12^iv^	3.3236
C9···H11A	2.7191	H9B···C13^iv^	3.3720
C9···H11B	3.3179	H9B···H1^iv^	2.5347
C9···H24A	3.3260	H9B···H25A^iv^	3.2878
C9···H24B	2.6728	H11A···O2^iv^	3.3418
C9···H24C	2.6682	H11A···C23^vi^	3.1848
C9···H25A	2.6849	H11A···H22B^vi^	3.2970
C9···H25B	2.6957	H11A···H22C^vi^	3.4992
C9···H25C	3.3375	H11A···H23A^vi^	2.5987
C11···H9A	3.3094	H11A···H23C^vi^	2.9148
C11···H9B	2.7892	H11A···H24A^xii^	2.9207
C11···H24A	2.6947	H11B···C24^xii^	3.3831
C11···H24B	3.3354	H11B···H11B^xiii^	3.5508
C11···H24C	2.6797	H11B···H23A^vi^	3.5428
C11···H25A	2.6776	H11B···H23C^vi^	3.5355
C11···H25B	3.3292	H11B···H24A^xii^	2.8542
C11···H25C	2.6784	H11B···H24C^ii^	3.2402
C12···H1	2.7208	H11B···H24C^xii^	3.1172
C12···H9B	3.3688	H17···O1^iv^	2.8952
C12···H25A	2.7712	H17···C14^iv^	3.4730
C12···H25C	3.4135	H17···C25^i^	3.2327
C12···H14	3.31 (3)	H17···H9A^ix^	3.5637
C13···H9A	3.2125	H17···H21^iv^	3.3260
C13···H9B	3.0200	H17···H22A^vi^	3.5506
C13···H11A	2.9274	H17···H24B^ix^	3.1705
C13···H11B	3.3065	H17···H25A^i^	2.8640
C13···H25A	2.9519	H17···H25B^i^	2.8858
C13···H14	3.02 (3)	H17···H25C^i^	3.4576
C13···H15	3.15 (3)	H17···H14^iv^	3.0895
C14···H21	2.7346	H18···O3^i^	3.4484
C15···H1	3.2578	H18···C8^i^	3.4780
C15···H17	2.6261	H18···C20^iv^	3.4206
C15···H21	2.6687	H18···C21^iv^	3.1313
C15···H23B	3.3580	H18···C25^i^	3.3155
C16···H18	3.2625	H18···H9A^ix^	3.4535
C16···H20	3.2668	H18···H9A^i^	3.3955
C16···H14	2.60 (3)	H18···H20^iv^	3.3044
C17···H21	3.2216	H18···H21^iv^	2.7628
C17···H15	2.60 (3)	H18···H25A^i^	2.6772
C18···H20	3.2365	H18···H25B^i^	3.1027
C19···H17	3.2011	H20···F1^x^	3.0448
C19···H21	3.1964	H20···O3^ix^	3.5868
C20···H18	3.2361	H20···C6^ix^	3.5732
C21···H17	3.2251	H20···C19^x^	3.4586
C21···H14	2.67 (3)	H20···C20^x^	3.1976
C21···H15	3.33 (3)	H20···H6A^ix^	3.5224
C22···H4A	2.6483	H20···H6B^ix^	2.8574
C22···H4B	2.7210	H20···H18^ii^	3.3044
C22···H6A	2.7709	H20···H20^x^	2.6610
C22···H6B	2.5677	H21···C17^ii^	3.4376
C22···H23A	2.6688	H21···C18^ii^	3.1224
C22···H23B	3.3055	H21···C22^iii^	3.5200
C22···H23C	2.6427	H21···H4A^iii^	3.4827
C23···H4A	3.3211	H21···H17^ii^	3.3260
C23···H4B	2.6184	H21···H18^ii^	2.7628
C23···H6A	2.5950	H21···H22A^iii^	2.7368
C23···H6B	3.3338	H21···H22B^iii^	3.4731
C23···H22A	3.3113	H21···H22C^vi^	3.3746
C23···H22B	2.6714	H22A···C16^viii^	3.3468
C23···H22C	2.6239	H22A···C17^viii^	3.1996
C24···H9A	2.7833	H22A···C18^viii^	3.3064
C24···H9B	2.5485	H22A···C19^viii^	3.5298
C24···H11A	2.6092	H22A···C21^viii^	3.5814
C24···H11B	2.7298	H22A···H17^viii^	3.5506
C24···H25A	3.3190	H22A···H21^vii^	2.7368
C24···H25B	2.6590	H22A···H14^vii^	2.9242
C24···H25C	2.6697	H22B···O2^vii^	2.6029
C25···H9A	2.5878	H22B···C14^vii^	3.3846
C25···H9B	3.3230	H22B···H1^vii^	3.3533
C25···H11A	3.3249	H22B···H11A^viii^	3.2970
C25···H11B	2.6250	H22B···H21^vii^	3.4731
C25···H24A	2.6618	H22B···H14^vii^	2.6050
C25···H24B	2.6684	H22C···C15^viii^	3.3203
C25···H24C	3.3184	H22C···C16^viii^	3.0316
H1···H14	2.3060	H22C···C17^viii^	3.5190
H1···H15	3.5473	H22C···C21^viii^	3.1694
H4A···H6B	2.6867	H22C···H4A^iv^	3.5817
H4A···H22A	2.5078	H22C···H11A^viii^	3.4992
H4A···H22B	2.8608	H22C···H21^viii^	3.3746
H4A···H22C	3.5429	H22C···H14^viii^	3.5369
H4A···H23A	3.5130	H23A···O2^vii^	3.2088
H4A···H23B	3.5669	H23A···C11^viii^	3.4709
H4B···H22A	3.0561	H23A···C24^ix^	3.2261
H4B···H22B	2.5231	H23A···H11A^viii^	2.5987
H4B···H22C	3.5691	H23A···H11B^viii^	3.5428
H4B···H23A	2.4253	H23A···H24A^ix^	2.4997
H4B···H23B	2.8818	H23A···H24B^ix^	3.1267
H4B···H23C	3.5018	H23A···H25C^ix^	3.2102
H6A···H22A	3.0515	H23B···O1^iv^	3.0772
H6A···H22C	2.6270	H23B···C24^ix^	3.4164
H6A···H23A	3.4987	H23B···H24A^ix^	3.0844
H6A···H23B	2.7933	H23B···H24B^ix^	2.8856
H6A···H23C	2.4373	H23B···H25B^ix^	3.2722
H6B···H22A	2.3597	H23B···H25C^ix^	3.4810
H6B···H22B	3.4599	H23C···O1^iv^	3.0534
H6B···H22C	2.8171	H23C···C3^iv^	3.5247
H6B···H23B	3.5981	H23C···C4^iv^	3.1830
H6B···H23C	3.5109	H23C···C11^viii^	3.5698
H9A···H24B	2.6192	H23C···H4A^iv^	3.1875
H9A···H24C	3.0991	H23C···H4B^iv^	2.5189
H9A···H25A	2.7968	H23C···H11A^viii^	2.9148
H9A···H25B	2.4202	H23C···H11B^viii^	3.5355
H9A···H25C	3.4894	H23C···H24A^ix^	3.3030
H9B···H11A	2.6795	H23C···H24B^ix^	3.4221
H9B···H24A	3.4538	H24A···C11^xii^	3.3105
H9B···H24B	2.7544	H24A···C23^xi^	3.1156
H9B···H24C	2.3679	H24A···C24^xii^	3.3320
H9B···H25B	3.4901	H24A···H11A^xii^	2.9207
H11A···H24A	2.8602	H24A···H11B^xii^	2.8542
H11A···H24B	3.4971	H24A···H23A^xi^	2.4997
H11A···H24C	2.4211	H24A···H23B^xi^	3.0844
H11A···H25A	3.5841	H24A···H23C^xi^	3.3030
H11A···H25C	3.5165	H24A···H24A^xii^	2.8015
H11B···H24A	2.5642	H24A···H24C^xii^	3.0825
H11B···H24B	3.5944	H24B···C17^xi^	3.3980
H11B···H24C	3.0192	H24B···C23^xi^	3.3204
H11B···H25A	2.8774	H24B···C25^iv^	3.3827
H11B···H25B	3.5111	H24B···H17^xi^	3.1705
H11B···H25C	2.4423	H24B···H23A^xi^	3.1267
H17···H18	2.3213	H24B···H23B^xi^	2.8856
H17···H15	2.3945	H24B···H23C^xi^	3.4221
H20···H21	2.3081	H24B···H25A^iv^	2.8715
H21···H14	2.1556	H24B···H25C^iv^	3.0227
H21···H15	3.5935	H24C···O2^iv^	2.8628
H22A···H23A	3.5554	H24C···C12^iv^	3.2611
H22A···H23C	3.5135	H24C···C25^iv^	3.4616
H22B···H23A	2.5079	H24C···H11B^iv^	3.2402
H22B···H23B	3.5474	H24C···H11B^xii^	3.1172
H22B···H23C	2.9393	H24C···H24A^xii^	3.0825
H22C···H23A	2.9083	H24C···H25A^iv^	2.9694
H22C···H23B	3.4988	H24C···H25C^iv^	3.1036
H22C···H23C	2.4290	H25A···C17^v^	3.2675
H23B···H15	2.7912	H25A···C18^v^	3.1618
H24A···H25A	3.5416	H25A···C24^ii^	3.3600
H24A···H25B	2.9248	H25A···H9B^ii^	3.2878
H24A···H25C	2.4867	H25A···H17^v^	2.8640
H24B···H25A	3.5415	H25A···H18^v^	2.6772
H24B···H25B	2.4823	H25A···H24B^ii^	2.8715
H24B···H25C	2.9505	H25A···H24C^ii^	2.9694
H24C···H25B	3.5378	H25B···O1^xi^	3.5309
H24C···H25C	3.5428	H25B···C15^xi^	3.3106
H14···H15	2.71 (4)	H25B···C16^xi^	3.2531
F1···H4A^i^	2.6922	H25B···C17^v^	3.5667
F1···H6A^ix^	3.2287	H25B···C21^xi^	3.5623
F1···H6B^i^	2.8599	H25B···H17^v^	2.8858
F1···H20^x^	3.0448	H25B···H18^v^	3.1027
O1···H6A^ii^	3.0975	H25B···H23B^xi^	3.2722
O1···H17^ii^	2.8952	H25C···O1^xi^	3.5911
O1···H23B^ii^	3.0772	H25C···C24^ii^	3.4827
O1···H23C^ii^	3.0534	H25C···H17^v^	3.4576
O1···H25B^ix^	3.5309	H25C···H23A^xi^	3.2102
O1···H25C^ix^	3.5911	H25C···H23B^xi^	3.4810
O1···H15^ii^	2.74 (3)	H25C···H24B^ii^	3.0227
O2···H4B^iii^	3.0419	H25C···H24C^ii^	3.1036
O2···H9B^ii^	3.0258	H14···C17^ii^	3.54 (3)
O2···H11A^ii^	3.3418	H14···C22^iii^	3.19 (3)
O2···H22B^iii^	2.6029	H14···H17^ii^	3.0895
O2···H23A^iii^	3.2088	H14···H22A^iii^	2.9242
O2···H24C^ii^	2.8628	H14···H22B^iii^	2.6050
O3···H1^iv^	3.5083	H14···H22C^vi^	3.5369
O3···H18^v^	3.4484	H15···O1^iv^	2.74 (3)
O3···H20^xi^	3.5868	H15···H1^iv^	3.5543
			
C7—O3—C8	117.86 (16)	C5—C4—H4A	108.816
C2—C1—C13	109.06 (17)	C5—C4—H4B	108.809
C2—C1—C14	112.04 (15)	H4A—C4—H4B	107.675
C13—C1—C14	110.00 (17)	C5—C6—H6A	108.873
C1—C2—C3	118.84 (18)	C5—C6—H6B	108.870
C1—C2—C7	122.35 (18)	C7—C6—H6A	108.879
C3—C2—C7	118.81 (18)	C7—C6—H6B	108.878
O1—C3—C2	121.1 (2)	H6A—C6—H6B	107.714
O1—C3—C4	121.5 (3)	C8—C9—H9A	108.964
C2—C3—C4	117.4 (2)	C8—C9—H9B	108.967
C3—C4—C5	113.74 (19)	C10—C9—H9A	108.960
C4—C5—C6	108.50 (17)	C10—C9—H9B	108.949
C4—C5—C22	111.7 (2)	H9A—C9—H9B	107.764
C4—C5—C23	108.13 (18)	C10—C11—H11A	108.599
C6—C5—C22	110.10 (19)	C10—C11—H11B	108.596
C6—C5—C23	109.69 (17)	C12—C11—H11A	108.585
C22—C5—C23	108.70 (18)	C12—C11—H11B	108.593
C5—C6—C7	113.47 (18)	H11A—C11—H11B	107.567
O3—C7—C2	122.94 (18)	C1—C14—H14	119.2 (16)
O3—C7—C6	110.85 (17)	C15—C14—H14	115.5 (16)
C2—C7—C6	126.20 (19)	C14—C15—H15	117.5 (14)
O3—C8—C9	110.75 (17)	C16—C15—H15	115.4 (14)
O3—C8—C13	122.79 (18)	C16—C17—H17	119.438
C9—C8—C13	126.46 (18)	C18—C17—H17	119.442
C8—C9—C10	113.10 (19)	C17—C18—H18	120.662
C9—C10—C11	108.21 (16)	C19—C18—H18	120.663
C9—C10—C24	109.18 (19)	C19—C20—H20	121.137
C9—C10—C25	110.37 (17)	C21—C20—H20	121.142
C11—C10—C24	110.12 (18)	C16—C21—H21	118.893
C11—C10—C25	109.78 (19)	C20—C21—H21	118.900
C24—C10—C25	109.18 (17)	C5—C22—H22A	109.474
C10—C11—C12	114.68 (17)	C5—C22—H22B	109.466
O2—C12—C11	121.55 (19)	C5—C22—H22C	109.466
O2—C12—C13	120.7 (2)	H22A—C22—H22B	109.474
C11—C12—C13	117.8 (2)	H22A—C22—H22C	109.478
C1—C13—C8	122.58 (18)	H22B—C22—H22C	109.470
C1—C13—C12	119.06 (18)	C5—C23—H23A	109.470
C8—C13—C12	118.28 (19)	C5—C23—H23B	109.469
C1—C14—C15	125.1 (2)	C5—C23—H23C	109.469
C14—C15—C16	126.9 (2)	H23A—C23—H23B	109.474
C15—C16—C17	120.00 (19)	H23A—C23—H23C	109.481
C15—C16—C21	122.54 (19)	H23B—C23—H23C	109.464
C17—C16—C21	117.45 (18)	C10—C24—H24A	109.469
C16—C17—C18	121.1 (2)	C10—C24—H24B	109.475
C17—C18—C19	118.7 (3)	C10—C24—H24C	109.473
F1—C19—C18	119.2 (3)	H24A—C24—H24B	109.474
F1—C19—C20	117.9 (3)	H24A—C24—H24C	109.474
C18—C19—C20	122.8 (3)	H24B—C24—H24C	109.463
C19—C20—C21	117.7 (3)	C10—C25—H25A	109.471
C16—C21—C20	122.2 (2)	C10—C25—H25B	109.473
C2—C1—H1	108.563	C10—C25—H25C	109.459
C13—C1—H1	108.543	H25A—C25—H25B	109.473
C14—C1—H1	108.553	H25A—C25—H25C	109.466
C3—C4—H4A	108.819	H25B—C25—H25C	109.484
C3—C4—H4B	108.810		
			
C7—O3—C8—C9	−172.44 (13)	O3—C8—C9—C10	159.36 (13)
C7—O3—C8—C13	7.8 (3)	O3—C8—C13—C1	4.2 (3)
C8—O3—C7—C2	−7.0 (3)	O3—C8—C13—C12	−179.08 (14)
C8—O3—C7—C6	172.08 (13)	C9—C8—C13—C1	−175.50 (15)
C2—C1—C13—C8	−15.0 (3)	C9—C8—C13—C12	1.2 (3)
C2—C1—C13—C12	168.36 (14)	C13—C8—C9—C10	−20.9 (3)
C13—C1—C2—C3	−164.10 (14)	C8—C9—C10—C11	44.1 (2)
C13—C1—C2—C7	15.7 (3)	C8—C9—C10—C24	163.94 (14)
C2—C1—C14—C15	44.9 (3)	C8—C9—C10—C25	−76.05 (19)
C14—C1—C2—C3	73.9 (2)	C9—C10—C11—C12	−52.5 (3)
C14—C1—C2—C7	−106.30 (19)	C24—C10—C11—C12	−171.74 (16)
C13—C1—C14—C15	−76.6 (3)	C25—C10—C11—C12	68.0 (2)
C14—C1—C13—C8	108.27 (19)	C10—C11—C12—O2	−145.70 (18)
C14—C1—C13—C12	−68.4 (2)	C10—C11—C12—C13	35.6 (3)
C1—C2—C3—O1	−2.8 (3)	O2—C12—C13—C1	−10.2 (3)
C1—C2—C3—C4	174.82 (14)	O2—C12—C13—C8	172.99 (17)
C1—C2—C7—O3	−5.9 (3)	C11—C12—C13—C1	168.47 (15)
C1—C2—C7—C6	175.25 (14)	C11—C12—C13—C8	−8.3 (3)
C3—C2—C7—O3	173.98 (15)	C1—C14—C15—C16	175.85 (18)
C3—C2—C7—C6	−4.9 (3)	C14—C15—C16—C17	−173.4 (2)
C7—C2—C3—O1	177.32 (16)	C14—C15—C16—C21	5.1 (4)
C7—C2—C3—C4	−5.0 (3)	C15—C16—C17—C18	177.44 (18)
O1—C3—C4—C5	−146.87 (19)	C15—C16—C21—C20	−177.67 (18)
C2—C3—C4—C5	35.5 (3)	C17—C16—C21—C20	0.9 (3)
C3—C4—C5—C6	−53.4 (2)	C21—C16—C17—C18	−1.1 (3)
C3—C4—C5—C22	−174.90 (15)	C16—C17—C18—C19	0.5 (4)
C3—C4—C5—C23	65.5 (2)	C17—C18—C19—F1	−179.2 (2)
C4—C5—C6—C7	43.3 (2)	C17—C18—C19—C20	0.4 (4)
C22—C5—C6—C7	165.83 (17)	F1—C19—C20—C21	178.92 (19)
C23—C5—C6—C7	−74.6 (2)	C18—C19—C20—C21	−0.7 (4)
C5—C6—C7—O3	165.12 (14)	C19—C20—C21—C16	0.0 (4)
C5—C6—C7—C2	−15.9 (3)		

Symmetry codes: (i) *x*+1, −*y*+1/2, *z*+1/2; (ii) *x*−1, *y*, *z*; (iii) −*x*+1, *y*+1/2, −*z*+1/2; (iv) *x*+1, *y*, *z*; (v) *x*−1, −*y*+1/2, *z*−1/2; (vi) −*x*+2, *y*+1/2, −*z*+1/2; (vii) −*x*+1, *y*−1/2, −*z*+1/2; (viii) −*x*+2, *y*−1/2, −*z*+1/2; (ix) *x*, −*y*+1/2, *z*+1/2; (x) −*x*+2, −*y*+1, −*z*+1; (xi) *x*, −*y*+1/2, *z*−1/2; (xii) −*x*+2, −*y*+1, −*z*; (xiii) −*x*+1, −*y*+1, −*z*.

### Hydrogen-bond geometry (Å, º)

**Table:**

*D*—H···*A*	*D*—H	H···*A*	*D*···*A*	*D*—H···*A*
C22—H22*B*···O2^vii^	0.96	2.60	3.533 (4)	163

Symmetry code: (vii) −*x*+1, *y*−1/2, −*z*+1/2.
